# Unwelcome Guest: Airborne Staph in Homes

**Published:** 2006-12

**Authors:** Carol Potera

*Staphylococcus aureus*, one of the most prevalent causes of infections of the blood, skin, soft tissue, and lower respiratory tract, spreads through close contact with contaminated people and surfaces. Although a few studies hint that airborne transmission of the microbe may be involved in hospital infections, no studies have examined indoor levels of *S. aureus* outside of a hospital setting. The first study to monitor *S. aureus* bioaerosols in residences shows that strains of the bacterium are common inhabitants of indoor and outdoor air **[*EHP* 114:1859–1864; Gandara et al.]**. Moreover, indoor strains are particularly resistant to commonly prescribed antibiotics.

During March, April, and May 2006, researchers cultured *S. aureus* from bioaerosol samples collected at 24 one-story homes in El Paso, Texas. They treated the bacterial colonies with three common antibiotics—ampicillin, penicillin, and cefaclor—to assess drug resistance.

All the indoor samples contained airborne *S. aureus*, as did nearly half of the outdoor samples. *S. aureus* levels inside the homes averaged 15.39 colony-forming units (CFU) per m^3^ air, and outdoor samples averaged 12.63 CFU per m^3^. About half the indoor samples were resistant to ampicillin, 60% were resistant to penicillin, and 13% were resistant to cefaclor. Samples of *S. aureus* collected outside the homes proved more susceptible to antibiotic killing, with 34% resisting ampicillin, 42% resisting penicillin, and 14% resisting cefaclor. In addition, about 14% of all *S. aureus* samples showed multidrug resistance, meaning the sample withstood both cefaclor and either penicillin or ampicillin. No other investigators have measured household levels of aerosolized *S. aureus* resistant to antibiotics, so the results have no basis for comparison.

The health consequences of living with *S. aureus* bioaerosols were not assessed in this study. The researchers plan to evaluate health risks associated with bacterial bioaerosols, such as whether elevated levels of drug-resistant *S. aureus* in indoor air parallel increases in community-acquired infections—that is, infections acquired outside a hospital setting.

The prevalence of drug-resistant *S. aureus* infections continues to rise, and rather than being confined largely to hospitals, such infections are increasing in the community, particularly in children. These new findings suggest that residential exposure to aerosolized *S. aureus* and a possible link to community-acquired *S. aureus* infections deserve further study.

## Figures and Tables

**Figure:**
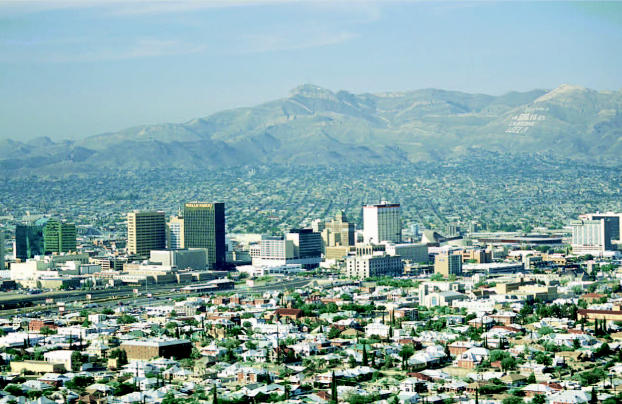
Down in the west Texas town A study in El Paso, Texas, shows evidence of antibiotic-resistant *Staphylococcus aureus* within residences.

